# Effect of Extrusion Temperature and Feed Moisture Content on the Microstructural Properties of Rice-Flour Pellets and Their Impact on the Expanded Product

**DOI:** 10.3390/foods11020198

**Published:** 2022-01-12

**Authors:** Yadira Zambrano, Ingrid Contardo, María Carolina Moreno, Pedro Bouchon

**Affiliations:** 1 Department of Chemical and Bioprocess Engineering, Faculty of Engineering, Pontificia Universidad Católica de Chile, Macul 6904411, Chile; yzambrano@uc.cl (Y.Z.); icontardo@uandes.cl (I.C.); mcmoreno@gmail.com (M.C.M.); 2 Laboratorio de Investigación e Ingeniería Biopolímeros (BiopREL), Escuela de Nutrición y Dietética, Facultad de Medicina, Universidad de los Andes, Monseñor Álvaro del Portillo 12.455, Las Condes 7620001, Chile

**Keywords:** 3G snacks, extrusion, rice, X-ray, micro-CT, microstructure

## Abstract

Extrusion can lead to an expanded product or to a slightly expanded pellet, known as a third-generation (3G) snack. In this case, expansion occurs subsequently, in an independent thermal device (e.g., oven), out of the extruded pellet. During both processes, several structural changes occur which are linked to processing conditions, including cooking temperature, screw speed, formulation, and initial moisture content. However, a clear relationship between processing variables and the structure of pellets and expanded products has not yet been identified. Accordingly, this work aimed to study the effect of extrusion temperature (110, 135, and 150 °C) and moisture content (27, 29, and 31%) in rice-flour pellets and their microwave expansion, through a microstructural approach using micro-CT. The results showed that the lowest moisture content (27%) and the highest extrusion temperature (150 °C) led to the highest pellet volume and the highest wall thickness, which in turn led to the highest expansion after microwave heating (50 s, 800 W). Interestingly, no significant differences were observed when analyzing the ratio between the volume of the expanded products and the volume of the pellet (~2.4) when using the different processing conditions.

## 1. Introduction

Extrusion, a high-temperature (e.g., 180–200 °C) and short-time process, is a suitable technology for snack production, as it enables a continuous and efficient system that ensures a uniform final product [1,2]. Twin-screw extruders are preferred over single-screw types, due to the improved processing control and wide range of materials that can be processed (e.g., viscous, sticky) [3,4]. Some important variables that influence the characteristics of an extruded product include screw configuration, screw speed, extrusion temperature and, certainly, the mix that is fed (i.e., ingredients and moisture content). Raw materials usually have high starch content due to their high water-absorption capacity and associated thermal transitions (gelatinization and retrogradation), which define the expansion and the final texture of the extrudates [5,6]. Accordingly, rice flour has become an appealing ingredient due its high starch content (~77%) [7], which allows for highly expanded products [8], but also, because it is gluten-free, hypoallergenic, and easy to digest [2,7,9,10,11].

Depending on the formulation and the processing conditions, the extruded product can undergo expansion either directly or indirectly. Direct expansion is the most common form [12], and refers to the immediate and final expansion of the product when it comes out of the extruder’s die exit, leading to a second-generation snack (2G Snack). In contrast, indirect expansion occurs after the extrusion process, in an independent thermal device (e.g., microwave oven), providing an expanded third-generation snack (3G Snack) out of a minimally expanded pellet that is produced in the extruder [8,13,14]. The advantages of 3G snacks over their 2G counterparts include an extended shelf life, a lower storage volume, and the possibility for companies to enter the extrudate market without the need to acquire the technology, only focusing on the expansion of acquired pellets [8,14]. 

Various authors have studied the effect of different extrusion parameters over the expansion of 2G products. Chanvrier et al. [15] and Philipp et al. [16] concluded that the initial water content is inversely proportional to the expansion, as previously observed [4,17,18]. It has also been determined that high extrusion temperatures enable the transformation of the raw material into a plasticized mass, helping to decrease the processing time [19,20], and that high screw speeds lead to a greater expansion as the intensity of the mechanical treatment increases, producing a greater macromolecular degradation of the ingredients and a decrease in the viscosity of the melted mass [16,21]. Accordingly, 3G snacks are prepared using a mix with higher initial water content (25–35% v/s 12–20%), and are processed at lower extrusion temperatures (100–150 °C v/s 150–200 °C), using moderate screw speeds (100–250 rpm v/s up to 400 rpm), compared to 2G snacks [1,22,23].

However, scientific studies on 3G snacks are limited. Camacho-Hernández et al. [24] determined that an optimum expansion of the extruded pellet, after subsequent heating, occurred when producing blue-corn and corn-starch pellets using a maximum extruding temperature ranging between 120 and 126 °C. They attributed this effect to the reduction in viscosity generated by a higher fragmentation of starch molecules that occurs at temperatures above 120 °C. Accordingly, Delgado-Nieblas et al. [14] concluded that higher expansion values of 3G pellets manufactured with yellow-corn and winter-squash flour using low initial moisture content and high temperatures (within the low-temperature range used in 3G snacks) were obtained due to improved gelatinization. For their part, Tovar-Jiménez et al. [25] showed that higher expansion values in the orange by-products of 3G snacks were obtained as feed moisture content decreased and the extruding temperature increased, within a low-temperature range. These results are consistent with the findings of Aguilar-Palazuelos et al. [26], who established that the extrusion temperature is an important factor for starch modification as excessive starch degradation tends to decrease subsequent expansion. Overall, these studies show the relationship between some extrusion parameters and the expanded pellet. However, no deep relationships between the characteristics of the pellet and the subsequent expanded product, together with their relationship with processing conditions, have been identified.

Most of the critical transformations in foods occur in the micrometer range and a major challenge consists in developing non-invasive microscopy techniques for the purpose of observation [27]. In this regard, X-ray microtomography (micro-CT) offers important advantages, as it practically requires no sample preparation, enabling a high resolution (on a micrometer scale) and spatial configuration of the inner structure. Its principle is based on the contrast observed in the image of a sample produced by the variations in the intensity of the X-ray attenuation and, therefore, it is particularly suited to characterize porous materials [21,28,29]. Accordingly, micro-CT has been used to analyze the porosity of extrudates [15,16,21,29]. For instance, Chanvrier et al. [15] and Babin et al. [29] found that extrudates with low porosity are obtained when processing mixtures with high initial moisture content in products based on rice and corn flour, respectively. Conversely, Philipp et al. [16] demonstrated that formulations containing high protein content result in structures with smaller pores and less expansion, whereas Robin et al. [21] found that an increase in extrusion temperature and screw speed, maintaining a constant bran concentration, increases the number of pores, while this number decreases when increasing the initial moisture content of the mix. Overall, these authors have quantified the expansion out of the extrusion process. However, focus on the relationship between the structure of extruded pellets and their subsequent expansion has yet to be developed.

Accordingly, the objective of this study was to assess the effect of the feed moisture content and the extrusion temperature on the microstructural characteristics of rice-flour pellets and their microwave expansion, through a microstructural approach using micro-CT image analysis.

## 2. Materials and Methods

### 2.1. Materials and Sample Preparation

Rice flour was used throughout this work (Extrumol Ltd., Santiago, Chile) which had an approximate water content of 11 ± 1% (the exact water content was determined individually for each sample), along with 87.7% available carbohydrates, 7.5% proteins, 2.8% fiber, 1.6% lipids, and 0.5% ash (dry basis, obtained from the manufacturer).

Mixes with different moisture contents were prepared by adding distilled water to the rice flour to yield formulations with 27%, 29%, and 31% moisture (wet basis), respectively, based on preliminary experiments. To do so, the rice flour was weighed on an electronic scale and homogenized with a food mixer for 1 min (5K5SS, KitchenAid, St. Joseph, MI, USA). Subsequently, the water was added continuously at room temperature for 1 min and mixing was continued for an additional 4 min. These samples were fed into the extruder.

### 2.2. Moisture Content

The moisture content of the rice flour, the mixes, and the pellets was determined by drying in a forced-air oven at 105 °C for 24 h (to constant mass), according to AOAC (1995).

### 2.3. Rheological Behavior of the Samples 

Dynamic oscillatory rheological measurements of the samples to be fed into the extruder were performed in a rheometer (Discovery, TA Instruments, New Castle, DE, USA), following the methodology explained by Ahmed et al. [30] and Min et al. [31], with some modifications. The geometry of the measuring system consisted of a Peltier steel plate with a 40 mm parallel plate diameter. Before rheological measurements, the sample was mixed with distilled water using a 1:0.5 (*w*/*w*) ratio and was allowed to rest for 30 min for relaxation. The mix was placed in a 2 mm gap between the parallel plates and the sample perimeter was covered with a thin layer of silicone oil to prevent the edges of the sample from drying. First, a strain sweep test (0.01 to 15%) was performed at a frequency of 10 rad/s at a controlled temperature (25 °C) to identify the linear viscoelasticity zone [32,33]. Frequency sweep tests (0.1 to 10 rad/s) were then performed at 25 °C at a strain of 0.1% to ensure a linear viscoelastic regime [10]. Finally, temperature sweep tests from 25 °C to 95 °C were performed in triplicate at a strain of 0.1% and a frequency of 10 rad/s to determine the storage modulus (G’) and the loss modulus (G”), as a function of temperature, to attain a closer understanding of the behavior of the samples during extrusion. All determinations were carried out in triplicate.

To obtain a complementary approximation to comprehend the behavior of the flow when the mix is heated inside a barrel and forced to flow through the die, the melt flow rate (MFR) was analyzed. The MFR of the samples at different moisture contents (27%, 29%, and 31%) was measured with a plastometer (LMI5500, Dynisco Instruments, Franklin, MA, USA). The plastometer consists of a heated cylinder at a standardized temperature, a piston with a standardized load, and a cutter [34,35]. The MFR was determined according to procedure B described in ASTM D1238, with some modifications. The sample was mixed with distilled water using a 1:0.8 (*w*/*w*) ratio, to obtain an excess of water. The samples were hydrated at room temperature for 15 min before feeding them into the equipment. Thereafter, each sample was fed into the cylinder at 95 °C and after 9 min a 5 kg load was applied with a piston. All samples were replicated, and the reported values are the average of three measurements expressed in g/10 min, which corresponds to the grams of sample that are forced to flow out of the equipment with a standardized load in a 10 min interval [36].

### 2.4. Extrusion Parameters

**Figure 1:**
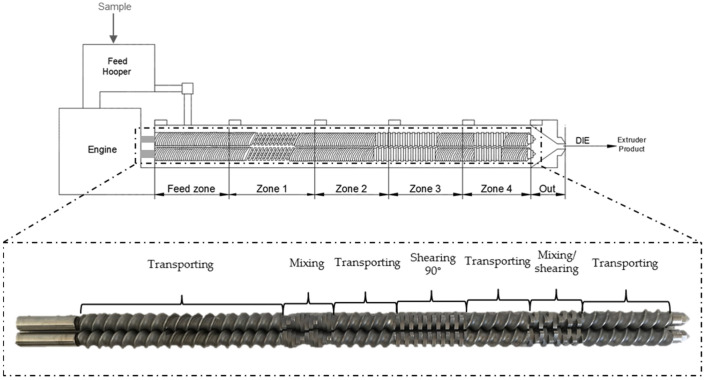
Diagram of the twin-screw extruder, showing the temperature zones and the configuration of the screws (which are shown one above the other, to facilitate visualization).

The samples were extruded using a co-rotating twin-screw extruder (Model 16 mm, Gülnar Makina, Kayseri, Turkey). The barrel was 40 cm long, the screw had a diameter of 16 mm, and the die had a diameter of 2 mm. The screw configuration from feed to die is shown in Figure 1 and had the following arrangement: one 1” conveying screw, three 2” conveying screws, three 30° forwarding paddles, one 2” conveying screw, four 90° kneading paddles, one 2” conveying screw, three 60° kneading paddles, one 1” conveying screw, and 2” conveying screws. The sequence remained constant in all experiments and was set based on preliminary tests and according to previous studies [37,38,39], which enhanced the homogenization and mix of the raw materials, allowing the dough to flow without sticking or burning.

The extruder had six barrel zones with independent temperatures: the first two temperatures were maintained at 40 °C and 80 °C, respectively; the third zone was set at 100 °C, 115.5 °C, or 120 °C, depending on the extrusion temperature (the maximum temperature of the process), which was set in the fourth zone, either at 110 °C, 135 °C, or 150 °C, whereas the last two zones were maintained at 80 °C [15,25,40,41]. The extrusion temperature, the most relevant temperature in the process, will be referred to as such throughout the text. The screw speed was maintained at 100 rpm to decrease the pressure, minimizing the expansion [17]. The pellets that were obtained from the extrusion process had an average cross-sectional diameter of ~4.3 mm and were cut at 25 rpm, collected, allowed to cool, packaged in a triple laminated aluminum bag with minimum headspace, and held at room temperature (~20 °C) before further analysis [42]. 

As pellets may be stored for long periods of time before their expansion, their changes after 1-, 7-, and 30-days’ production were analyzed and their expansion was examined after 30-days’ storage.

### 2.5. Texture and Expansion Ratio of the Pellet

The texture of the pellet was determined using an Instron Universal testing machine (Model 4206, Instron Corp., Norwood, MA, USA) to determine the hardness (N), according to Philipp et al. [16]. The maximum force required to break the sample was considered as hardness and corresponded to the higher peak of the force–deformation curve. A Warner–Bratzler shear blade was used at a descendent rate of 1 mm/s. The samples were placed individually at their central cross-section on the platform. Hardness was measured after 1, 7, and 30 days from the manufacture of the pellets. All experiments were carried out in triplicate, and each value corresponded to the mean of 15 measurements. 

The expansion ratio of the pellet (ERP) was defined as the ratio between the diameter of the cross-section of the pellet and the diameter of the die [43,44], where the cross-sectional diameter of the sample was measured using a caliper. All experiments were carried out in triplicate, and each value corresponded to the mean of 42 random measurements. 

### 2.6. Water Activity 

The water activity (a_w_) of the pellets was measured in triplicate 1, 7, and 30 days after their manufacture, using Novasina (NOVASINA Company, Lachen, Switzerland).

### 2.7. Microwave Expansion Process

Since pellets may be stored for long periods of time before their final sale, they were expanded after 30 days of storage. Fourteen pellets of each bath were placed in the center of the turntable of a commercial microwave oven (Samsung, model ME73M, 800 W, Samsung, Seremban, Malaysia) and were heated during a time-lapse of 40, 50, or 60 s. The expanded products had a cylindrical shape with an average cross-sectional diameter of ~10 mm. 

### 2.8. Texture of the Expanded Product

The texture of the expanded products was determined using a texture analyzer (TA.XT2, Stable Micro System Ltd., Godalming, UK), equipped with a 5 kg load cell. The hardness was measured using a three-point bending test [45]. The distance between the supports was 6 mm. The samples were placed individually at their central cross-section on the support, and the test speed was set at 1 mm/s. The highest peak of the force–deformation curve represented the resistance to breakage (N) of the sample. All experiments were carried out in triplicate, and each value corresponded to the mean of 15 measurements. 

### 2.9. Microstructural Analysis Using Micro-CT

After 30 days’ storage, the pellets and the expanded products were scanned using a high-resolution micro-CT (Skyscan 1272, version 1.1.17, Bruker Corp., Kontich, Belgium). For this purpose, the pellets were stuck on a sample holder and scanned. Thereafter, they were expanded by heating in the microwave for 50 s (the optimum heating time) and were scanned once again. All determinations were carried out in triplicate. 

#### 2.9.1. Image Acquisition and Reconstruction Process

Images were acquired using an X-ray beam at 35 kV at a constant current of 231 µA [46,47,48]. The optimum experimental conditions during image acquisition were set at an image pixel size of 7.5 μm, including 1344 × 2016 pixels per image. The samples were scanned over an interval of 0–180° with a rotation step of 0.2°. The exposure time was ~68 min per sample. No filter was used in the scanning.

The reconstruction of the dataset was performed using, on average, 1203 projection images, setting the contrast at 0–0.14 in pellets and at 0–0.15 in expanded products, using reconstruction software (NRecon v. 1.7.3, SkyScan, Kontich, Belgium). 

The parameters related to the reconstruction process included: post-alignment, beam-hardening correction, smoothing, and the reduction in ring artifacts. The beam-hardening and the smoothing were set up to 15% and 1, respectively, in pellets, and to 50% and 1, respectively, in expanded products. 

#### 2.9.2. Image Analysis

The reconstructed images were processed and analyzed using CTAn software (version 1.18.8.0, SkyScan, Kontich, Belgium). The volume of interest (VOI) of the pellets and the expanded products was selected from the middle part of the image, cutting out the top and bottom sections, so as to include relevant information and eliminate edge effects or artifacts.

Overall, the images were segmented and cleaned by eliminating rings and residuary artifacts. A multilevel (5) automatic thresholding was performed in pellets, whereas a global thresholding was applied to expanded products. Thereafter, custom processing tools, including despeckle, erosion, dilation, and ROI shrink-wrap functions, were used to obtain the best adjustment to the surface of the pellets and of the expanded products.

The images were quantified using 3D image analysis with a voxel size of 7.5 µm × 7.5 µm × 7.5 µm. The volume of the solid matrix (MV), the volume of the pores (PV), and the total volume (TV = MV + PV) were then determined. In addition, in expanded products, the structure thickness of the matrix and the structure thickness of the pores were determined, and were used as wall-thickness and pore-size descriptors. The structure thickness was obtained by identifying the medial axis of the structure through skeletonization and a sphere-fitting was applied to enclose the diameter of the largest sphere along this axis [49]. 

### 2.10. Statistical Analysis

Extrusion trials for all combinations (extrusion temperature and feed moisture content) were conducted in triplicate. For each replicate, the measurement of a_w_ was performed in triplicate, whereas the hardness of the pellets, the hardness of the expanded products, and the expansion ratio of the pellets were determined using 15, 15, and 42 replicates, respectively. The image analysis of pellets and expanded products was carried out in triplicate for each replicate. One-way variance analysis (ANOVA) was carried out to determine significant differences in normally distributed data, whereas the Kruskal–Wallis test was performed for non-normally distributed data, using Statgraphics Centurion XVI version 16.1.03 (Stat-Point Technologies Inc., Warrenton, VA, USA). The LSD method was used to determine the significant differences between the results with a confidence level of 95%. A correlation matrix was also calculated to study possible relationships between process parameters, along with the characteristics of pellets and expanded products, using XLSTAT Student 2020.4.1.1016 (XLSTAT by Addinsoft, New York, NY, USA). The expanded product properties were set as dependent variables and the pellet properties as quantitative variables, whereas qualitative variables were the extrusion temperature and the feed moisture content. This correlation was analyzed with a confidence level of 95% and a tolerance of 0.0001. Overall, results are expressed as a mean ± standard deviation or ± typical error.

## 3. Results and Discussion

First, the rheological behavior of the samples at different moisture contents (27%, 29%, and 31%) was analyzed to characterize the mix that was fed into the extruder, using two approaches. Thereafter, the textural and microstructural characteristics of the pellet and the expanded product were analyzed to understand possible relationships between the pellet and the resultant expanded product. To further comprehend this phenomenon, the microstructural properties of the expanded product were characterized and quantified using micro-CT image analysis. 

### 3.1. Rheological Properties of the Feeding Mix

**Figure 2:**
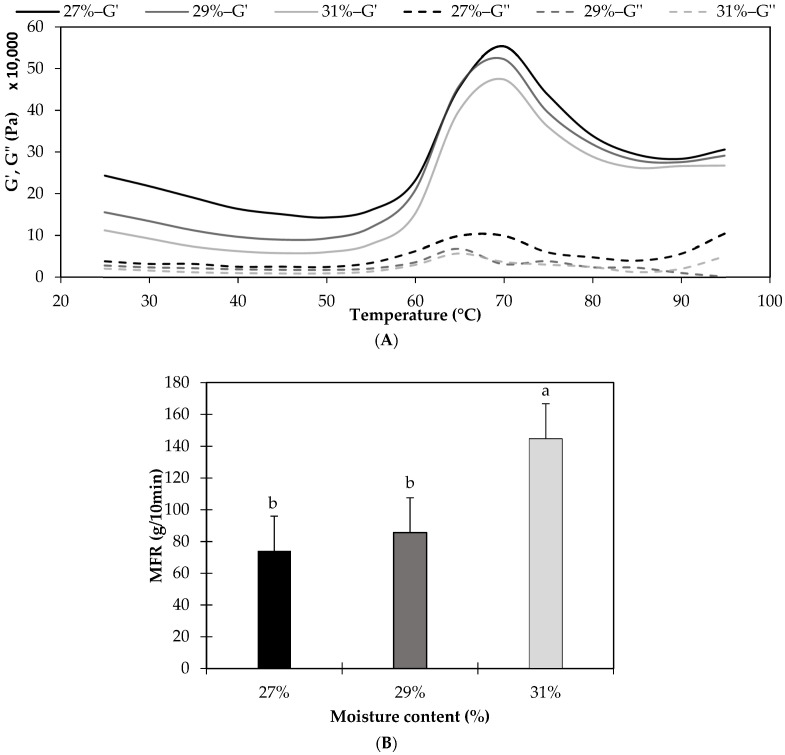
Rheology of the samples that were fed into the extruder, prepared with rice and 27, 29, or 31% water. (**A**) Storage moduli (G’) are shown in solid lines and Loss moduli (G”) in dotted lines; (**B**) Melt flow rate (MFR). Different letters show significant differences (*p* < 0.05). Data are means of three samples ± typical error.

Rheology is an instrumental analysis to understand the role of water in the mix that was fed into the extruder with different moisture levels (27%, 29%, and 31%) and increasing temperatures, to obtain an understanding of the behavior of the sample during the extrusion process. Results were analyzed in terms of the storage modulus (G’), which is the capacity of the sample to store energy and represents the elastic part of its viscoelastic behavior, and in terms of the loss modulus (G”), which is the capacity of the sample to lose energy and represents the viscous component of its viscoelastic behavior. These can be directly used as elasticity modulus and viscosity modulus, respectively, in case of a linear static analysis [50,51]. Figure 2A shows the influence of the moisture content on G’ and G” as a function of the sweep temperature (from 25 °C to 95 °C), where each curve was obtained from the average of three samples. Overall, it is possible to observe that all curves had a similar shape, in which the elastic behavior predominated during the whole temperature range, denoting a solid-like structure, which started to increase significantly at ~55 °C. Subsequently, G’ reached a maximum value at ~70 °C, a peak that could be linked to starch gelatinization. As reported, the gelatinization peak of starch in rice flour occurs at 70 °C [52], increasing the apparent viscosity of the dough [3,53]. As heating progressed, G’ decreased, probably due to the breakdown of the residual crystalline structure as temperature increased [54,55].

Regarding the effect of moisture, no significant differences (*p* > 0.05) were determined when comparing the samples with 27% and 29% moisture or when comparing the samples with 29% and 31% moisture. Significant differences (*p* < 0.05) were only found when comparing the samples with 27% and 31% moisture, and the highest mean values of G’ and G” were observed at the lowest moisture content (27%). This is consistent with other studies that have demonstrated that higher amounts of water result in lower G’ and G” values [10,54,55,56,57,58,59], as water may increase sample deformation, reducing granule–granule interactions, increasing granule hydration, and decreasing sample resistance and rigidity [54,56,58], which is a behavior that could well occur inside the extruder barrel. 

To obtain an understanding of the flow of the sample when heated inside a barrel and forced to flow through a die, the melt flow rate (MFR) was analyzed. The MFR measures how fast a thermoplastic material flows throughout a standardized hole under a specific temperature and standardized weight, and is inversely proportional to its viscosity [34,35,60]. Figure 2B shows the MFR of the sample at different moisture contents. Significant differences (*p* < 0.05) were found when comparing samples with 31% moisture and samples with 27% or 29% moisture, whereas no significant differences (*p* > 0.05) were found between the latter. Furthermore, samples with 31% moisture had the highest MFR, denoting an easier flow [61]. Overall, these results are consistent with those reported previously (Figure 2A) and reflect that an increase in moisture content may facilitate the flow of the sample, decreasing G’ and G”, a phenomenon that could well happen within the extruder barrel. 

### 3.2. Relationship between Processing Conditions and the Extruded Pellets

**Figure 3:**
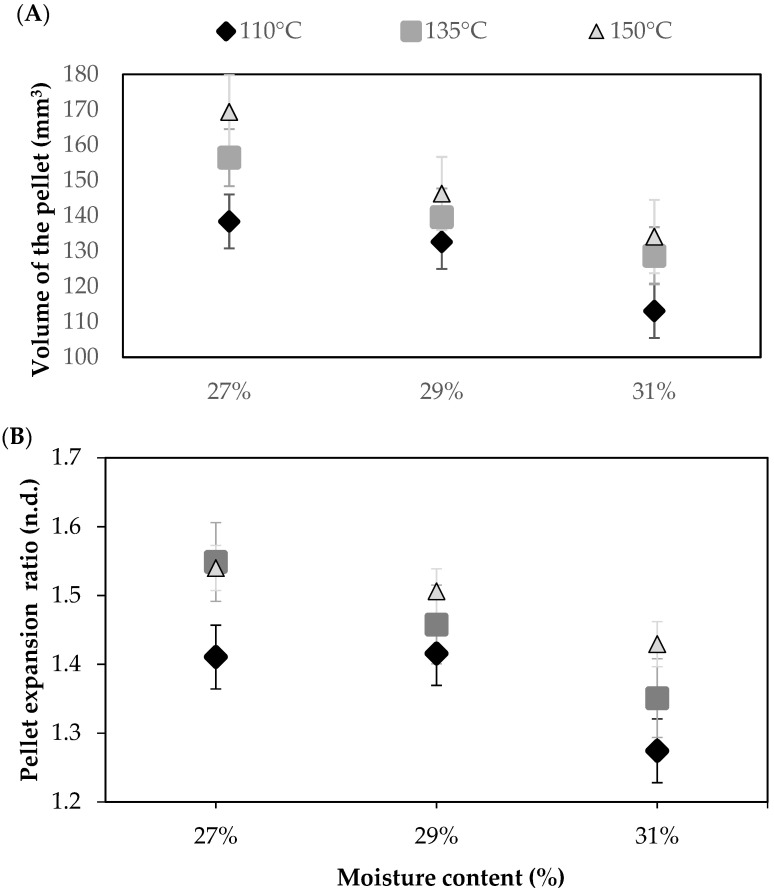
Microstructural characteristics of the pellets produced feeding a rice-flour mix with different moisture levels (27%, 29%, or 31%) and processed using different extrusion temperatures (110 °C, 135 °C, or 150 °C). (**A**) Volume of the pellet (VP). Results are expressed as mean (*n* = 3) ± typical error. (**B**) Expansion ratio of the pellet (ERP). Results are expressed as mean (*n* = 42) ± typical error.

The relationship between extrusion conditions and the structural characteristic of the pellet is of interest, as it may help understanding of the link between processing parameters and the expanded product. In this study, the volume of the pellet (VP) (obtained from micro-CT image analysis) and the expansion ratio of the pellet (ERP) (measured with a caliper) were considered. Figure 3A shows the relationship between the feed moisture content and VP at different extrusion temperatures. On the whole, the VP exhibited a significant increase (*p* < 0.05) when feed moisture content decreased, at a constant temperature. The only exception was at 110 °C, where no significant differences between samples prepared with 27% and 29% feed moisture content were determined. It is important to note that higher VP is mostly obtained at lower feed moisture contents. This increase in VP could be related to dough rheology, as previously observed by Singha et al. [61], who also found similar results when analyzing dried grains during extrusion as well as a lower viscosity with an increase in feed moisture content. A lower value of G’ and a higher value of MFR (Figure 2) were obtained for the higher feed moisture content (31%), which may translate into an increase in the flow of the melt as well as a reduction in the elastic component of its viscoelasticity, aspects that may facilitate the movement of the mix along the extruder barrel. This may reduce the friction between the screw, the extruder barrel, and the feed, decreasing heat transfer to the dough, and contributing to a lower expansion at the die. Cisneros and Kokini [62] studied the relationship between the barrel fill length and entrapment of air bubbles in the unexpanded extrudate. They showed that a reduction in the friction inside the extruder barrel with higher feed moisture contents, to avoid superheated dough at the die exit, decreased unwanted expansion. Similarly, Sandrin et al. [63] found that lubrication within the extruder decreased the friction and thus reduced the expansion of 2G extrudates. Finally, as pointed out by Dalbhagat et al. [57] and Maskan and Altan [17], high feed moisture contents reduce the viscoelasticity of the dough inside the extruder, resulting in a lower expansion. These results, which were obtained from micro-CT image analysis, were corroborated by measuring the ERP with a caliper. Figure 3B shows the relationship between the feed moisture content and ERP at different extrusion temperatures. These results showed the same tendency as that observed in Figure 3A and are similar to other studies [23,25], which had reported that an increase in feed moisture content reduces the expansion ratio.

The lowest VP or ERP values were obtained in pellets extruded using an extrusion maximum of 110 °C, and were significantly lower (*p* < 0.05) compared to those processed with an extrusion temperature of 150 °C, for all feed moisture contents. Even though VP and ERP mean values at 135 °C were higher, when compared to 110 °C, significant differences (*p* < 0.05) between these temperatures were only found at 27% and 31% feed moisture contents. Overall, VP and ERP values rose with an increase in extrusion temperature, which may thus enhance moisture evaporation at the die exit [19].

### 3.3. Influence of Processing Conditions on Textural Properties of Pellets

Pellets have a long shelf life so they can be stored for long periods of time, facilitating their handling by occupying small volumes [8,64]. In fact, in this study, the water activity of the different samples after extrusion did not show significant differences and was on average ~0.67, providing an appropriate shelf life. Accordingly, evaluation of the resistance of pellets to breakage over time is important. In this work, this was measured as hardness, which was expressed as the maximum breaking force (N) obtained from the force–deformation curve [65]. 

**Figure 4:**
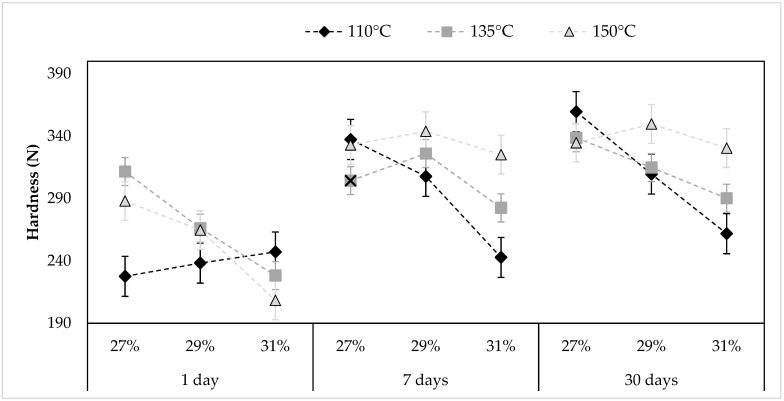
Hardness of the pellets produced feeding a rice-flour mix with different moisture levels (27%, 29%, or 31%) and processed using different extrusion temperatures (110 °C, 135 °C, or 150 °C), 1, 7, or 30 days after extrusion. Data show the mean of fifteen samples ± typical error.

Figure 4 shows the variation in hardness of the pellets over time, 1, 7, and 30 days after extrusion for all the studied conditions. Overall, the mean values of hardness of pellets after 1-day extrusion had the lowest hardness compared to those stored for 7 or 30 days after extrusion. After 1-day storage, no significant differences were observed in hardness between samples prepared using different feed moisture contents processed at an extrusion temperature of 110 °C. Regarding samples processed at extrusion temperatures of 135 °C and 150 °C, a decrease in hardness was observed when the feed moisture content was 31%, which was significantly lower (*p* < 0.05) than those obtained in samples prepared with a 27% or 29% feed moisture content. Although the hardness mean values of samples with 29% feed moisture content were lower than those obtained in samples with a 27% feed moisture content, a significant difference between these two moisture contents was observed only at 135 °C, after 1-day storage. Furthermore, no significant differences (*p* > 0.05) were observed in hardness when comparing samples processed at extrusion temperatures of 135 °C or 150 °C for the different feed moisture contents, after 1 day. Thus, when processing mixes at temperatures higher than 110 °C, the resistance to breakage after 1-day extrusion depended mainly on the feed moisture content. 

No significant differences (*p* > 0.05) were found when comparing the hardness of the pellets after 7 and 30 days’ storage, at any extrusion temperature, in samples prepared with a feed moisture content of 29% and 31%. That is, hardness was not affected during this time interval in this set of samples. Some significant differences were only observed in samples prepared with 27% feed moisture content, extruded at extrusion temperatures of 110 or 135 °C. However, said differences were minor (<11%). These results show that the main changes in hardness occurred between 1 and 7 days, and could be due to a rapid molecular rearrangement at the beginning of storage, at a constant moisture level [66]. 

### 3.4. Relationship between Processing Conditions, Pellets, and Expanded Products 

**Figure 5:**
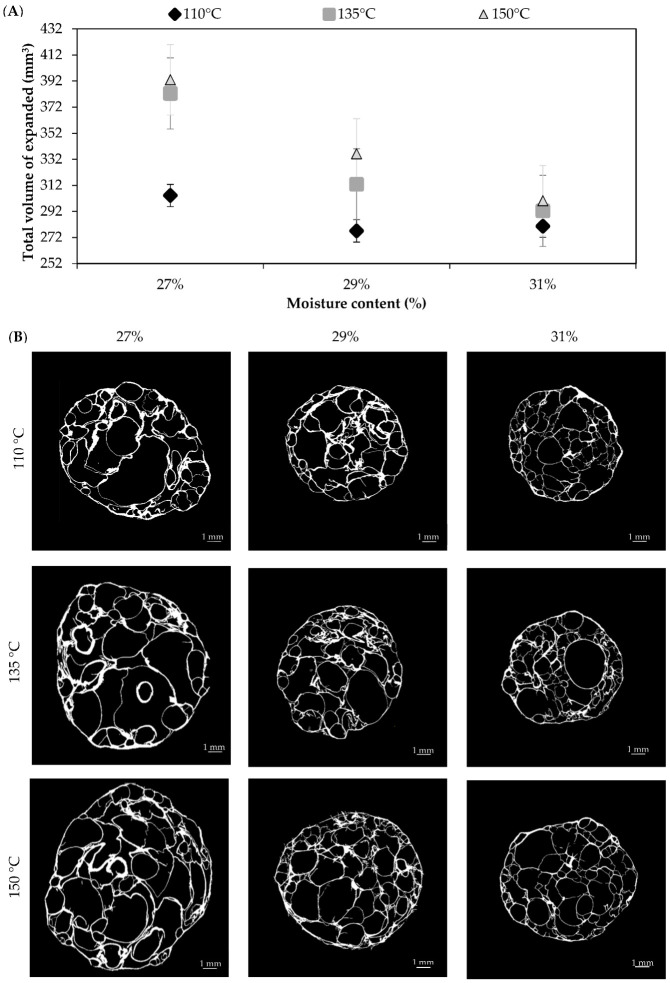
(**A**) Total volume of the expanded products obtained after microwaving (800 W) pellets for 50 s, which were produced feeding a rice-flour mix with different moisture levels (27%, 29%, or 31%) and processed using different extrusion temperatures (110 °C, 135 °C, or 150 °C). Results are expressed as mean (*n* = 3) ± typical error. (**B**) Cross-section X-ray image of the matrix volume of expanded products (MVE). The scale bar represents 1 mm.

Optimum microwave expansion was obtained after 50 s, as this microwave time led to complete expansion of the pellets without their suffering from burning. Figure 5A shows the effect of the feed moisture content at different extrusion temperatures on the total volume of the expanded products (TVE) that were obtained after microwaving for 50 s. Generally, the lowest feed moisture content (27%) produced a higher TVE. Regarding the extrusion temperature, no significant effect (*p* > 0.05) was obtained in the TVE when comparing the same value of feed moisture content between the highest extrusion temperatures (135 °C and 150 °C). However, a significant effect (*p* < 0.05) occurred when comparing the lowest extrusion temperature (110 °C) with the other two extrusion temperatures (135 °C and 150 °C) at feed moisture contents of 27% and 29%. In samples prepared with 31% feed moisture, no significant differences (*p* > 0.05) were determined between the different temperatures. However, it is important to note that the highest mean values of TVE (Figure 5A) were obtained at the highest temperature (150 °C) and the lowest feed moisture content (27%). These results could be related to the rheology of the dough (G’, G”, MFR) during the extrusion. High feed moisture content produces a decrease in G’ and G”, which are the elastic and viscous parts of the viscoelasticity behavior of the dough, respectively. This would allow the dough to flow more easily, as explained in Section 3.3, resulting in a less expanded pellet, which achieves a lower volume after microwave heating. Interestingly, no significant differences were obtained when analyzing the ratio between the volume of the expanded products and the volume of the pellet (TVE/VP in Table 1). This means that no matter the initial moisture content and extrusion temperature that were used, a similar relative expansion was obtained (~2.4 on average), whereas the final dimensions of the expanded product were directly linked to the conditions used. According to Lee et al. [67], a greater viscoelasticity allows for the formation of stable air cells quite easily in the expanded products, thus aiding higher expansion. Due to the fact that sufficient viscoelasticity enhances the capacity of starch to retain gas, this allows vapor pressure to accumulate without disrupting cell structure. On the other hand, low viscoelasticity forms small voids instead of air cells [67]. 

To further understand and visualize the effect of feed moisture content and extrusion temperature in the microstructure of the expanded product, the hardness, the porosity, the wall thickness, the mean pore size, and the pore-size distribution of the expanded products were studied. Figure 5B shows a representative cross-sectional X-ray image of the highly porous structure of the expanded products (MVE) at different feed moisture contents and extrusion temperatures. The wall of the expanded products, represented in white, enclosed a wide range of pores of different sizes and shapes, and these were quantified by micro-CT. Table 1 shows the mean porosity (%), the wall thickness (μm), the pore size (μm), and the hardness (N) of the expanded pellets, after microwave heating. 

No significant differences (*p* > 0.05) in hardness were obtained between the different expanded samples. Additionally, no correlations were found between the hardness and the porosity, which corresponds to the percentage of the pore volume over the total volume of the expanded product. The mean wall thickness and the mean pore size were obtained as the weighted average of the structure-thickness distribution of the matrix volume and the pore volume of the expanded product, respectively, and were obtained through the quantification of micro-CT 3D images. Although some pellets had a higher expansion, no significant differences were obtained between the different extrusion temperatures or feed moisture contents in the porosity (%) or in the mean wall thickness of the expanded products. To obtain a further understanding of the relationship between the different parameters, the correlations between the different microstructural features presented in Table 1 were analyzed. Among these, a negative correlation (r = −0.85) between porosity and wall thickness was obtained, indicating that expanded products with higher porosity tended to have thinner walls, regardless of the conditions that were used (extrusion temperature and feed moisture content). 

**Figure 6:**
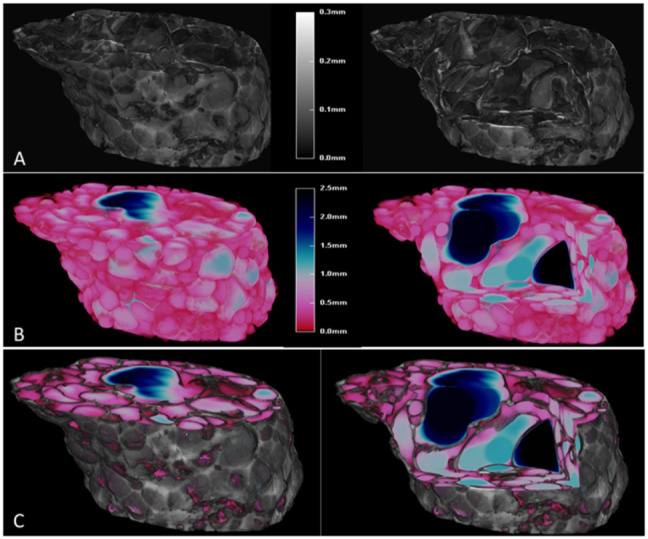
The 3D micro-CT images of the expanded product obtained after microwaving (800 W) for 50 s, pellets that were obtained using an extrusion temperature of 110 °C and feeding rice flour with 27% moisture content, without a virtual cut (left) and with a virtual cut (right). (**A**) Grayscale 3D volume-rendering image, where the grayscale reflects the structure-thickness distribution of the matrix, representing the wall thickness. (**B**) Color-coded structure-thickness distribution of the pores ranging from 0 to 2.5 mm, representing the air pores. (**C**) Overlay of wall thickness and pores.

**Figure 7:**
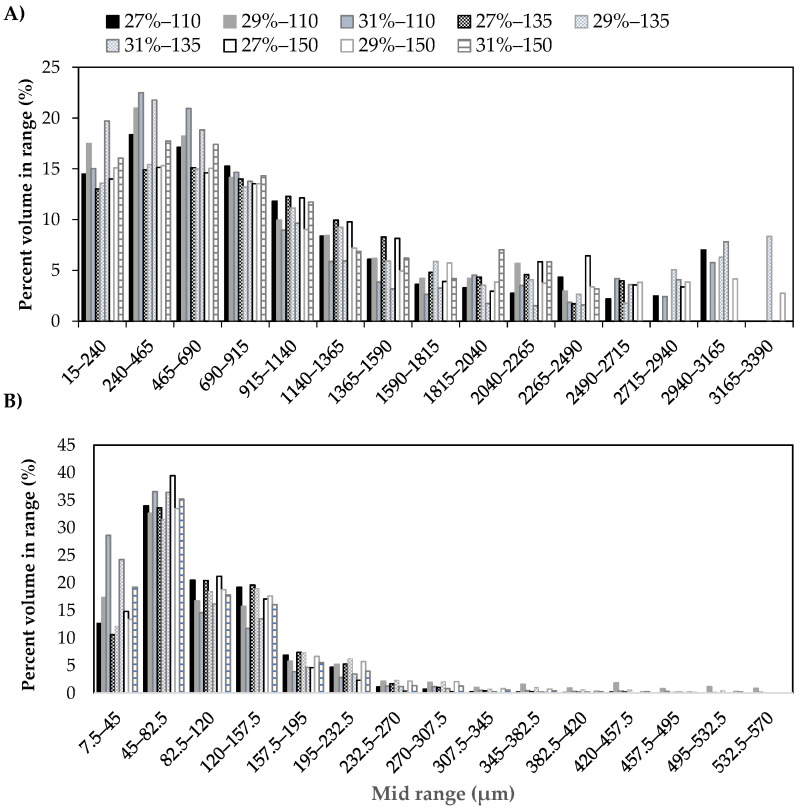
Structure-thickness distribution of (**A**) pore size and (**B**) wall size of the expanded products obtained after microwaving (800 W) pellets for 50 s, produced feeding a rice-flour mix with different moisture levels (27%, 29%, or 31%) and processed using different extrusion temperatures (110 °C, 135 °C, or 150 °C).

To better understand and visualize the microstructural properties of the expanded product, its structure-thickness distribution was analyzed. A 3D representation is shown in Figure 6. Each color represents a specific size range of the wall thickness (Figure 6A) or pore size (Figure 6B), according to the color-bar legend, while Figure 6C shows an overlay of the wall thickness and the pores. The structure-thickness distribution of the matrix is represented as a grayscale in Figure 6A and refers to the wall thickness. For its part, Figure 6B shows the structure-thickness distribution of the pores and is linked to the pore size. The smallest pores (<0.5 mm) are represented in pink and the largest pores (>2.0 mm) are represented by colors ranging from blue to black. It can be seen that there are mostly small pores and only a few big pores appeared. These bigger pores could correspond to the initial pores within the pellet, known as nucleation sites (trapped air in the matrix) [23]. In addition, a quantification of structure-thickness distribution of the matrix and the pores was performed using micro-CT image analysis. Figure 7 is a quantitative method that represents the structure-thickness distribution of the pores as a pore-size distribution (Figure 7A) and the structure-thickness distribution of the expanded matrix as a wall size distribution (Figure 7B). The results show the percentage of pores or wall volume within the mid-range (μm). Overall, the pore-size distribution is similar when comparing the samples prepared with different initial feed moisture contents and processed using different extrusion temperatures, and is mostly comprised of small pore sizes (< 915 μm, Figure 7A) and thinner walls (<82.5 μm, Figure 7B). Similar values of mean pore size in expanded products have been reported by Chanvrier et al. [68], in corn (150–600 μm) and wheat extrudates (600–1200 μm) using 20% feed moisture content and an extrusion temperature of 135 °C. Additionally, the results obtained from micro-CT image analysis are consistent with those reported by Gimeno et al. [69], who found small pores and thinner walls in extrudates made of corn flour with xanthan gum. In this study, the majority of pores (77–83%) presented a size range between 15 and 915 μm (Figure 7A), and a higher percentage within this range was obtained in samples prepared with a feed moisture content of 31%. This could be related to the lower mean expanded volumes that were obtained at this moisture content (Figure 5A). On the other hand, the wall thickness (Figure 7B) was manly distributed between 7.5 and 157.5 μm. The extruded product obtained with a feed moisture content of 27% and an extrusion temperature of 150 °C showed the highest percentage (~40%), being samples which in turn had the highest volume (Figure 5A). 

## 4. Conclusions

Image analysis using micro-CT allowed an adequate characterization of the microstructural properties of extruded 3G pellets and expanded products obtained through different processing conditions, allowing the quantification of their porosity, wall thickness, pore size, total volume, pore volume, and matrix volume. A similar tendency was obtained between the results of the quantification of the pellet volume with micro-CT and the results of the expansion ratio of the pellets. It is important to highlight that micro-CT analysis did not show significant differences in the ratio between the volume of the expanded products and the volume of the pellet, meaning a similar relative expansion regardless of the extrusion parameters (moisture content and extrusion temperature). Overall, the highest pellet volume was obtained at the lowest feed moisture content (27%) and the highest extrusion temperature (150 °C). This behavior was related to the elastic and viscous parts of the viscoelastic behavior of the sample inside the extruder barrel, suggesting that lower feed moisture content increases the friction and temperature, making it more difficult for the sample to move along the extruder barrel, to finally obtain a higher expansion at the die. This behavior could be also linked to the glass transition temperature of the samples, an aspect that could be addressed in future studies. Accordingly, pellets obtained at 27% of feed moisture content and 150 °C extrusion temperature led to the highest wall thickness and volume of the expanded product during microwave heating, probably due to their capacity to withstand the vapor pressure without disrupting the cell structure. However, no significant differences were obtained in hardness of the expanded products. 

## Figures and Tables

**Table 1:** Microstructural properties and hardness of the expanded products obtained after microwaving (800 W) pellets for 50 s, which were produced feeding a rice-flour mix with different moisture levels (27%, 29%, or 31%) and processed using different extrusion temperatures (110 °C, 135 °C, or 150 °C). TVE refers to the volume of the expanded product and VP to the volume of the pellet. Data are means ± confidence interval at 95%. Different letters denote significant differences (*p* < 0.05).

Extrusion Conditions		TVE/VP (n.d.)	Porosity (%)	Wall Thickness (μm)	Pore Size (μm)	Hardness (N)
110 °C	27%	2.3 ± 0.2 ^a^	79.4 ± 2.3 ^a^	97.1 ± 11.4 ^a^	808.7 ± 0.1 ^ab^	25.8 ± 3.1 ^a^
	29%	2.3 ± 0.3 ^a^	77.9 ± 4.6 ^a^	110.3 ± 37.2 ^a^	705.4 ± 0.2 ^a^	29.1 ± 3.8 ^a^
	31%	2.6 ± 0.4 ^a^	83.5 ± 2.8 ^a^	76.5 ± 16.5 ^a^	722.3 ± 0.1 ^a^	23.0 ± 2.6 ^a^
135 °C	27%	2.5 ± 0.2 ^a^	81.8 ± 2.3 ^a^	93.9 ± 12.2 ^a^	870.9 ± 0.1 ^b^	27.9 ± 2.8 ^a^
	29%	2.3 ± 0.2 ^a^	79.1 ± 3.6 ^a^	106.9 ± 21.0 ^a^	935.9 ± 0.1 ^b^	27.2 ± 3.7 ^a^
	31%	2.6 ± 0.3 ^a^	80.6 ± 4.3 ^a^	81.1 ± 16.4 ^a^	694.4 ± 0.1 ^a^	24.7 ± 2.9 ^a^
150 °C	27%	2.5 ± 0.2 ^a^	81.3 ± 1.8 ^a^	91.5 ± 9.3 ^a^	879.1 ± 0.1 ^ab^	27.8 ± 3.5 ^a^
	29%	2.4 ± 0.2 ^a^	79.2 ± 4.0 ^a^	101.7 ± 19.2 ^a^	982.7 ± 0.2 ^b^	25.9 ± 3.9 ^a^
	31%	2.4 ± 0.2 ^a^	80.5 ± 3.3 ^a^	89.0 ± 16.6 ^a^	769.1 ± 0.1 ^a^	25.2 ± 2.8 ^a^

## References

[1] Harper J.M. (1994). The Technology of Extrusion Cooking.

[2] Bryant R.J., Kadan R.S., Champagne E.T., Vinyard B.T., Boykin D. (2001). Functional and digestive characteristics of extruded rice flour. Cereal Chem..

[3] de Pinho Ferreira Guine R., Correia P.M. (2014). Engineering Aspects of Cereal and Cereal-Based Products.

[4] Rahman S., Ahmed J. (2012). Handbook of Food Process Design.

[5] Tovar-Jiménez X., Aguilar-Palazuelos E., Gómez-Aldapa C.A., Caro-Corrales J. (2016). Microstructure of a third generation snack manufactured by extrusion from potato starch and orange vesicle flour. J. Food Process. Technol..

[6] Castellanos-Gallo L., Galicia-García T., Estrada-Moreno I., Mendoza-Duarte M., Márquez-Meléndez R., Portillo-Arroyo B., Soto-Figueroa C., Leal-Ramos Y., Sanchez-Aldana D. (2019). Development of an Expanded Snack of Rice Starch Enriched with Amaranth by Extrusion Process. Molecules.

[7] Ding Q.-B., Ainsworth P., Tucker G., Marson H. (2005). The effect of extrusion conditions on the physicochemical properties and sensory characteristics of rice-based expanded snacks. J. Food Eng..

[8] Panak Balentić J., Babić J., Jozinović A., Ačkar Đ., Miličević B., Muhamedbegović B., Šubarić D. (2018). Production of third-generation snacks. Croat. J. Food Sci. Technol..

[9] Kadan R.S., Pepperman A.B. (2002). Physicochemical properties of starch in extruded rice flours. Cereal Chem..

[10] Moreira R., Chenlo F., Torres M.D. (2013). Rheology of gluten-free doughs from blends of chestnut and rice flours. Food Bioprocess Technol..

[11] Sharma C., Singh B., Hussain S.Z., Sharma S. (2017). Investigation of process and product parameters for physicochemical properties of rice and mung bean (Vigna radiata) flour based extruded snacks. J. Food Sci. Technol..

[12] Riaz M.N. (2000). Extruders in Food Applications.

[13] Ruiz-Armenta X.A., de Jesús Zazueta-Morales J., Aguilar-Palazuelos E., Delgado-Nieblas C., López-Diaz A., Camacho-Hernández I.L., Gutiérrez-Dorado R., Martínez-Bustos F. (2017). Effect of extrusion on the carotenoid content, physical and sensory properties of snacks added with bagasse of naranjita fruit: Optimization process. CyTA-J. Food.

[14] Delgado-Nieblas C., Aguilar-Palazuelos E., Gallegos-Infante A., Rocha-Guzmán N., Zazueta-Morales J., Caro-Corrales J. (2012). Characterization and optimization of extrusion cooking for the manufacture of third-generation snacks with winter squash (Cucurbita moschata D.) flour. Cereal Chem..

[15] Chanvrier H., Nordström Pillin C., Vandeputte G., Haiduc A., Leloup V., Gumy J.C. (2015). Impact of extrusion parameters on the properties of rice products: A physicochemical and X-ray tomography study. Food Struct..

[16] Philipp C., Oey I., Silcock P., Beck S.M., Buckow R. (2017). Impact of protein content on physical and microstructural properties of extruded rice starch-pea protein snacks. J. Food Eng..

[17] Maskan M., Altan A. (2011). Advances in Food Extrusion Technology.

[18] Steel C.J., Leoro M.G.V., Schmiele M., Ferreira R.E., Chang Y.K., El-Sonbati A. (2012). Thermoplastic extrusion in food processing. Termoplastic Elastomers.

[19] Guy R. (2001). Extrusion Cooking: Technologies and Applications.

[20] Karwe M.V., Barbosa-Cánovas G.V. (2009). Food Extrusion. Food Engineering-Volume III.

[21] Robin F., Dubois C., Pineau N., Schuchmann H.P., Palzer S. (2011). Expansion mechanism of extruded foams supplemented with wheat bran. J. Food Eng..

[22] Huber G.R., Rokey G.J. (1990). Extruded Snacks. Snack Food.

[23] Moraru C.I., Kokini J. (2003). Nucleation and expansion during extrusion and microwave heating of cereal foods. Compr. Rev. Food Sci. Food Saf..

[24] Camacho-Hernández I.L., Zazueta-Morales J.J., Gallegos-Infante J.A., Aguilar-Palazuelos E., Rocha-Guzmán N.E., Navarro-Cortez R.O., Jacobo-Valenzuela N., Gómez-Aldapa C.A. (2014). Effect of extrusion conditions on physicochemical characteristics and anthocyanin content of blue corn third-generation snacks. CyTA-J. Food.

[25] Tovar-Jiménez X., Caro-Corrales J., Gómez-Aldapa C.A., Zazueta-Morales J., Limón-Valenzuela V., Castro-Rosas J., Hernández-Ávila J., Aguilar-Palazuelos E. (2015). Third generation snacks manufactured from orange by-products: Physicochemical and nutritional characterization. J. Food Sci. Technol..

[26] Aguilar-Palazuelos E., de Jesús Zazueta-Morales J., Harumi E.N., Martínez-Bustos F. (2012). Optimization of extrusion process for production of nutritious pellets. Food Sci. Technol..

[27] Aguilera J.M. (2005). Why food microstructure?. J. Food Eng..

[28] Parada J., Aguilera J.M., Brennan C. (2011). Effect of guar gum content on some physical and nutritional properties of extruded products. J. Food Eng..

[29] Babin P., Della Valle G., Dendievel R., Lourdin D., Salvo L. (2007). X-ray tomography study of the cellular structure of extruded starches and its relations with expansion phenomenon and foam mechanical properties. Carbohydr. Polym..

[30] Ahmed J., Almusallam A.S., Al-Salman F., AbdulRahman M.H., Al-Salem E. (2013). Rheological properties of water insoluble date fiber incorporated wheat flour dough. LWT Food Sci. Technol..

[31] Min W., Yi L., Lijun W., Dong L. (2015). Effects of extrusion parameters on rheological properties, chromatism, protein solubility and microstructure of flaxseed-corn mixture Citation. Int. J. Agric. Biol. Eng..

[32] Letang C., Piau M., Verdier C. (1999). Characterization of wheat flour-water doughs. Part I: Rheometry and microstructure. J. Food Eng..

[33] Keentok M., Newberry M.P., Gras P., Bekes F., Tanner R.I. (2002). The rheology of bread dough made from four commercial flours. Rheol. Acta.

[34] Khan R.M., Mushtag A., Israr A., Nafees A. (2015). Comparative study for melt flow index of different microstructure polyethylene. Pakistan J. Eng. Appl. Sci..

[35] Mertz A.M., Mix A.W., Baek H.M., Giacomin A.J. (2013). Understanding Melt Index and ASTM D1238. J. Test. Eval..

[36] Kormin S., Kormin F., Beg H. (2019). Effect of plasticizer on physical and mechanical properties of ldpe/sago starch blend. J. Phys. Conf. Ser..

[37] Kartika A.I., Pontalier P.Y., Rigal L. (2006). Extraction of sunflower oil by twin screw extruder: Screw configuration and operating condition effects. Bioresour. Technol..

[38] Kirby A.R., Ollett A.-L., Parker R., Smith A.C. (1988). An experimental study of screw configuration effects in the twin-screw extrusion-cooking of maize grits. J. Food Eng..

[39] Nabar Y., Narayan R., Schindler M. (2006). Twin-screw extrusion production and characterization of starch foam products for use in cushioning and insulation applications. Polym. Eng. Sci..

[40] Gulati P., Weier S.A., Santra D., Subbiah J., Rose D.J. (2016). Effects of feed moisture and extruder screw speed and temperature on physical characteristics and antioxidant activity of extruded proso millet (*Panicum miliaceum*) flour. Int. J. Food Sci. Technol..

[41] Ghumman A., Kaur A., Singh N., Singh B. (2016). Effect of feed moisture and extrusion temperature on protein digestibility and extrusion behaviour of lentil and horsegram. LWT-Food Sci. Technol..

[42] Boischot C., Moraru C.I., Kokini J. (2003). Factors that influence the microwave expansion of glassy amylopectin extrudates. Cereal Chem..

[43] Fleischman E.F., Kowalski R.J., Morris C.F., Nguyen T., Li C., Ganjyal G., Ross C.F. (2016). Physical, textural, and antioxidant properties of extruded waxy wheat flour snack supplemented with several varieties of bran. J. Food Sci..

[44] Meng X., Threinen D., Hansen M., Driedger D. (2010). Effects of extrusion conditions on system parameters and physical properties of a chickpea flour-based snack. Food Res. Int..

[45] Robin F., Engmann J., Pineau N., Chanvrier H., Bovet N., Valle G. (2010). Della Extrusion, structure and mechanical properties of complex starchy foams. J. Food Eng..

[46] Contardo I., Bouchon P. (2018). Enhancing Micro-CT methods to quantify oil content and porosity in starch-gluten matrices. J. Food Eng..

[47] Gondek E., Jakubczyk E., Herremans E., Verlinden B., Hertog M., Vandendriessche T., Verboven P., Antoniuk A., Bongaers E., Estrade P. (2013). Acoustic, mechanical and microstructural properties of extruded crisp bread. J. Cereal Sci..

[48] Trater A.M., Alavi S., Rizvi S.S.H. (2005). Use of non-invasive X-ray microtomography for characterizing microstructure of extruded biopolymer foams. Food Res. Int..

[49] Bruker Introduction to Porosity Analysis—Method Note 59. www.brukersupport.com.

[50] Liu Y., Yu Y., Liu C., Regenstein J.M., Liu X., Zhou P. (2019). Rheological and mechanical behavior of milk protein composite gel for extrusion-based 3D food printing. LWT.

[51] Kristiawan M., Della Valle G., Kansou K., Ndiaye A., Vergnes B. (2019). Validation and use for product optimization of a phenomenological model of starch foods expansion by extrusion. J. Food Eng..

[52] Dautant F.J., Simancas K., Sandoval A.J., Müller A.J. (2006). Effect of temperature, moisture and lipid content on the rheological properties of rice flour. J. Food Eng..

[53] Aguilera J.M., Lillford P.J. (2008). Food Materials Science.

[54] Rolee A., LeMeste M. (1999). Effect of moisture content on thermomechanical behavior of concentrated wheat starch-water preparations. Cereal Chem..

[55] Kraus S., Enke N., Gaukel V., Schuchmann H.P. (2014). Influence of degree of gelatinization on expansion of extruded, starch-based pellets during microwave vacuum processing. J. Food Process Eng..

[56] Assifaoui A., Champion D., Chiotelli E., Verel A. (2006). Rheological behaviour of biscuit dough in relation to water mobility. Int. J. Food Sci. Technol..

[57] Dalbhagat C.G., Mahato D.K., Mishra H.N. (2019). Effect of extrusion processing on physicochemical, functional and nutritional characteristics of rice and rice-based products: A review. Trends Food Sci. Technol..

[58] Ditudompo S., Takhar P.S., Ganjyal G.M., Hanna M.A. (2013). The effect of temperature and moisture on the mechanical properties of extruded cornstarch. J. Texture Stud..

[59] Fitzgerald M.A., Martin M., Ward R.M., Park W.D., Shead H.J. (2003). Viscosity of rice flour: A rheological and biological study. J. Agric. Food Chem..

[60] Wang S., Capoen L., D’hooge D.R., Cardon L. (2018). Can the melt flow index be used to predict the success of fused deposition modelling of commercial poly (lactic acid) filaments into 3D printed materials?. Plast. Rubber Compos..

[61] Singha P., Muthukumarappan K., Krishnan P. (2018). Influence of processing conditions on apparent viscosity and system parameters during extrusion of distiller’s dried grains-based snacks. Food Sci. Nutr..

[62] Cisneros F.H., Kokini J. (2002). A generalized theory linking barrel fill length and air bubble entrapment during extrusion of starch. J. Food Eng..

[63] Sandrin R., Caon T., Zibetti A.W., de Francisco A. (2018). Effect of extrusion temperature and screw speed on properties of oat and rice flour extrudates. J. Sci. Food Agric..

[64] Aguilar-Palazuelos E., de Jesús Zazueta-Morales J., Martínez-Bustos F. (2006). Preparation of High-Quality Protein-Based Extruded Pellets Expanded by Microwave Oven. Cereal Chem. J..

[65] Aguilera J.M. (2013). Edible Structures: The Basic Science of What We Eat.

[66] Chung H.J., Lim S.T. (2004). Physical aging of glassy normal and waxy rice starches: Thermal and mechanical characterization. Carbohydr. Polym..

[67] Lee E.Y., Lim K.I., Lim J.K., Lim S.T. (2000). Effects of gelatinization and moisture content of extruded starch pellets on morphology and physical properties of microwave-expanded products. Cereal Chem..

[68] Chanvrier H., Jakubczyk E., Gondek E., Gumy J.-C. (2014). Insights into the texture of extruded cereals: Structure and acoustic properties. Innov. Food Sci. Emerg. Technol..

[69] Gimeno E., Moraru C.I., Kokini J.L. (2004). Effect of xanthan gum and CMC on the structure and texture of corn flour pellets expanded by microwave heating. Cereal Chem..

